# Model-Driven Impact Quantification of Energy Resource Redundancy and Server Rejuvenation on the Dependability of Medical Sensor Networks in Smart Hospitals

**DOI:** 10.3390/s22041595

**Published:** 2022-02-18

**Authors:** Francisco Airton Silva, Carlos Brito, Gabriel Araújo, Iure Fé, Maxim Tyan, Jae-Woo Lee, Tuan Anh Nguyen, Paulo Romero Martin Maciel

**Affiliations:** 1 Laboratory of Applied Research to Distributed Systems (PASID), Universidade Federal do Piauí (UFPI), Picos 64607-670, Brazil; faps@ufpi.edu.br (F.A.S.); carlosvictor@ufpi.edu.br (C.B.); gabrielholanda@ufpi.edu.br (G.A.); iuresf@gmail.com (I.F.); 2 Konkuk Aerospace Design-Airworthiness Research Institute (KADA), Konkuk University, Seoul 05029, Korea; 3 Department of Aerospace Information Engineering, Konkuk University, Seoul 05029, Korea; 4 Centro de Informática, Universidade Federal de Pernambuco, Recife 50740-560, Brazil; prmm@cin.ufpe.br

**Keywords:** Internet of Things (IoT), smart hospital, energy resources, availability, stochastic Petri net

## Abstract

The spread of the Coronavirus (COVID-19) pandemic across countries all over the world urges governments to revolutionize the traditional medical hospitals/centers to provide sustainable and trustworthy medical services to patients under the pressure of the huge overload on the computing systems of wireless sensor networks (WSNs) for medical monitoring as well as treatment services of medical professionals. Uncertain malfunctions in any part of the medical computing infrastructure, from its power system in a remote area to the local computing systems at a smart hospital, can cause critical failures in medical monitoring services, which could lead to a fatal loss of human life in the worst case. Therefore, early design in the medical computing infrastructure’s power and computing systems needs to carefully consider the dependability characteristics, including the reliability and availability of the WSNs in smart hospitals under an uncertain outage of any part of the energy resources or failures of computing servers, especially due to software aging. In that regard, we propose reliability and availability models adopting stochastic Petri net (SPN) to quantify the impact of energy resources and server rejuvenation on the dependability of medical sensor networks. Three different availability models (A, B, and C) are developed in accordance with various operational configurations of a smart hospital’s computing infrastructure to assimilate the impact of energy resource redundancy and server rejuvenation techniques for high availability. Moreover, a comprehensive sensitivity analysis is performed to investigate the components that impose the greatest impact on the system availability. The analysis results indicate different impacts of the considered configurations on the WSN’s operational availability in smart hospitals, particularly 99.40%, 99.53%, and 99.64% for the configurations A, B, and C, respectively. This result highlights the difference of 21 h of downtime per year when comparing the worst with the best case. This study can help leverage the early design of smart hospitals considering its wireless medical sensor networks’ dependability in quality of service to cope with overloading medical services in world-wide virus pandemics.

## 1. Introduction

The Internet of Medical Things (IoMT) has become an important computing paradigm in recent years due to the emergence of new diseases around the world. In IoMT, wireless medical devices and sensors are integrated and harmonized into a common network in smart hospitals [1]. IoMT is a medical monitoring system that provides continuous real-time monitoring and observation services to patients through wearable health sensors and devices with wireless body area network (WBAN), artificial intelligence (AI), and remote monitoring techniques [2]. With the functional advantages of IoMT, an early warning system equipped with real-time data collection and storage can do in-depth and rapid analysis to control the spread of infectious diseases and decrease the workload of healthcare services. Wireless sensor data generated by hardware devices, such as cell phones, are usually transmitted to a cloud/fog computing platform for decision making. Therefore, the operational continuity of the IoMT’s overall infrastructure, from its power supply network in remote areas to the IT systems of local hospitals, is essential in this period.

Smart hospital infrastructures have many functions that keep them running [3], such as (i) medical equipment (wireless sensors) for remote monitoring and remote diagnostic diagnostics; (ii) networked medical equipment (bracelet heart rate monitor wireless thermometer, blood glucose meter, etc.); (iii) network device (transmission media routers gateways, etc.); (iv) data (related to patient or staff information); and (v) buildings and facilities (some sensors are distributed throughout the hospital). The proper functioning and integration of these five macro-resources must be rigorously ensured and continuously maintained. Computer systems in hospitals often need to run as efficiently as possible with minimal latency. However, local servers alone are not enough to handle the large volumes of data generated during severe pandemic cases. Therefore, using not only edge servers but also cloud servers is imperative. Such a complex and distributed infrastructure involving sensors, actuators, and servers could not be missing any component. In some cases, failure of one component can lead to a complete failure of the patient monitoring system. Therefore, it is essential to carry out studies to ensure the maximum availability of such systems.

Without exception, IoMT is certainly prone to partial crashes and system crashes [4]. In the context of a pandemic, the ability to maintain 24/7 medical services from your IoMT is essential, but it is difficult due to the large number of data transactions that can lead to unexpected events, unexpected medical events, and even serious losses of life. The possibility of actual active incidents of IoMT in medical centers has been demonstrated in reports in practice. At LDS Hospital in Salt Lake City, Utah, USA, a computerized hospital information system called Health Assessment Through Logic Programming (HELP) handles 17,000 logins per day [5,6]. A survey of Electronic Medical Record (EMRD) downtime in a crowded urban emergency department from May 2016 to December 2017 in [7] found there was a total of more than *58 h of downtime*, and *12 episodes of EMRD* occurred during the study period with *5-h unpredictable intervals*. The EHR system at the National Institutes of Health Clinical Center (NIHCC) in the United States unexpectedly closed on 13 May 2010, resulting in all patients suddenly losing access to available clinical information, potentially affecting patient care and safety [8]. These factors require IoMT researchers to understand the nature of availability issues and their solutions through detailed modeling and system design evaluation before the system is deployed.

Assessing the availability of hospital computer systems is important but sometimes unfeasible to perform in real environments. Hospital computer systems are sensitive as they handle critical patient data. These systems and equipment usually have a high monetary cost, making on-site experiments unfeasible. Two aspects that initially directly impact the availability of smart hospitals are the electricity service quality provided to the hospital and issues related to the software system’s aging. If the hospital does not have electricity, its systems will be off. If software performance is affected by computational aging (memory leakage, for example), it will also cause general availability to drop somewhat. Thus, the question that guides this paper is: *What is the impact of using different energy sources and software rejuvenation methods on the availability of smart hospital computer systems?*

This work has two main focuses—energy issues and the smart hospital. The related work has focused on one of the two contexts in isolation. Oueida et al. [9] and Greco et al. [10] were the only works that focused on IoT. Araujo(b) et al. [11] was unique in the context of smart buildings. All experimental works in the energy context used reliability or availability metrics without directing them to the hospital context. Some smart hospitals performed availability analysis without observing energy issues and software aging. The works by Oueida et al. [9], Greco et al. [10], Chen et al. [12], and Araujo(a) et al. [13] used metrics more focused on the performance area. Therefore, to the best of our knowledge, our work is unique in exploring availability and reliability in the hospital context by looking at energy and software aging issues.

The availability analysis of hospital systems supported by IoMT is often unfeasible in real environments. Formal mathematical models such as queue networks, Markov chains, and Petri nets can be adopted at an early design stage or evaluate the complex configurations required in an operating system. This paper proposes a series of stochastic Petri net (SPN) models [14,15,16] to represent and evaluate a smart hospital architecture looking at energy issues and software aging. Petri net is a mathematical form based on probability theory that allows evaluating any system that undergoes some state change. SPN can represent synchronization, sequencing, parallelization, and concurrency, among other aspects of any distributed system. SPN has already been adopted in previous studies in the hospital context, however, without focusing on energy issues and software aging [17,18]. Therefore, the main contributions of this paper are:

- **Three availability SPN models to evaluate the availability feature of a smart hospital system**. The models may calculate the availability of the IoMT system. Two models are the extensions of the first one. Thus, the first model includes only energy resources with a power grid and a diesel power generator that delivers energy to the hospital. The second model includes a redundant point at the energy supply by including a solar system energy resource. The third model explores a rejuvenation strategy under some important model components. These three models can be the reality of a real smart hospital, and this work can provide these models to guide system designers to optimize their infrastructures, for example.
- **An SPN model that calculates the IoMT reliability**. Reliability is the probability that the system has performed its function up to a predetermined and uninterrupted time limit. We have varied a specific parameter related to the cloud aging time in the third proposed configuration model. Thus, experiments have shown that the cloud aging time impacts system reliability.
- **A sensitivity analysis of the base model**. The analysis has shown which components have the greatest impact on IoMT system availability. The power grid, for example, was the most impacting component in one of the IoMT system configurations. In other cases, the aging aspects of edge and cloud had a greater impact on the availability.

The rest of this paper is organized as follows: Section 2 presents related works. Section 3 presents an overview of the architecture of the modeled system. Section 4 presents the proposed SPN models, their functioning, and their peculiarities. Section 5 shows the results of the sensitivity analysis of the availability model. Section 6 presents the results for three case studies, which serve as a practical guide for a system administrator. Finally, Section 7 concludes this work and discusses possible future work.

## 2. Related Work

This section presents some related work with similar approaches or contexts to this work. Oueida et al. [9] proposed a resource preservation net (RPN) framework using Petri nets. The work presents a framework capable of generating non-consumable resource models that are theoretically described and validated. The work aims to measure some performance indicators of an intelligent hospital system with edge and cloud processing components. Among the performance metrics of the study, there is the patient’s length of stay (LoS), resources usage rate, and average waiting time. Santos et al. [17] propose analytical models of Petri nets and a reliability block diagram (RBD) to assess the availability of an intelligent health monitoring system that depends on edge, fog, and cloud infrastructures. Santos et al. [17] still use a multi-objective optimization algorithm (NSGA-II) to improve system availability, taking into account its cost as a limitation.

Greco et al. [10] propose a technological and architectural solution based on open source big data technologies to perform real-time data flow analysis on wearable sensors. The architecture proposed by the work comprises four layers: the sensing layer, the pre-processing layer, the cluster processing layer, and the persistence layer. Each layer’s performance analysis was performed to gauge each layer’s memory and CPU usage. Chen et al. [12] propose an Edge-Cognitive-Computing-based (ECC-based) smart-healthcare system. The system can monitor and analyze the status of patients using cognitive computing. Furthermore, the system can allocate resources according to the patient’s degree of risk. Experiments have shown that the system improves the user experience, optimizes resources, and increases patient survival chances in sudden emergencies.

Araujo(a) et al. [13] propose a high-level model capable of characterizing the behavior of an mHealth system. The objective of the work was to identify the probability of a system message being delivered in *t* time. The paper did not analyze availability, but some parts of the model were characterized as an availability model. Lisboa et al. [19] propose a patient monitoring architecture using sensors and cloud and fog processing. The work also presents a sensitivity analysis that identifies the components that most impact system availability. Santos et al. [20] also propose a monitoring architecture using cloud and fog. However, ref. [20] extends the idea proposed in [19] and adds a model that can calculate performance metrics and identify possible bottlenecks in the system.

Rodrigues et al. [18] propose models capable of calculating performance and availability metrics in a smart hospital system. The work presents a performance model capable of calculating Mean Response Time, Resource Utilization, and Discard. The work also presents an availability model and performs a sensitivity analysis on this model. The results show optimal settings for system performance and availability. The article by [18] is one of two works that this work extends. The study by Araujo(b) et al. [11] proposes an energy availability model for intelligent construction. The paper investigates the impact of different types of solar panel energy systems on the availability of smart buildings. In addition, the work also makes a cost comparison for the adopted energy systems.

Diaz et al. [21] developed a methodological strategy to improve the conditions of autonomous photovoltaic systems through reliability research in the laboratory and rural areas. Collins et al. [22] propose an RBD model capable of calculating failure rates for different components of large photovoltaic systems. The work results show the relationship between availability and reliability concerning system uptime. The work by Sayed et al. [23] proposes an analysis of the availability, reliability, and maintainability of a photovoltaic system. RBD can represent a myriad of possibilities in terms of component relationships or parameters. Finally, Cai et al. [24] propose a framework for evaluating the reliability of grid-connected photovoltaic systems with intermittent failures using Dynamic Bayesian Networks (DBNs). The work uses the framework to assess the availability and reliability of different photovoltaic systems. Table 1 displays related works. The works were classified considering seven aspects: context, metrics, evaluation method, whether it considered the use of energy, sensitivity analysis, availability, and reliability.

The first classification criterion is the work’s **context**. The context concerns the main theme of the work. This work has two main contexts: energy issues and smart hospitals. The other works focused on one of the two contexts in isolation. Oueida et al. [9], Santos et al. [17], and Greco et al. [10] were focused on IoT. Araujo(b) et al. [11] was unique in the context of smart buildings. This work is focused on both the context of smart hospitals and the context of energy supply.

The second classification criterion was the investigated **metric**. All works in the energy context used reliability or availability metrics without directing them to the hospital context. Some smart hospitals performed availability analysis without observing energy issues and software aging. The works by Oueida et al. [9], Greco et al. [10], Chen et al. [12], and Araujo(a) et al. [13] used metrics more focused on the performance area. Our work is unique in exploring availability and reliability in the hospital setting by looking at energy issues and software aging.

The third ranking criterion is the **evaluation method**. Analytical models were the most common method due to their practicality and agility in generating results. The most used analytical models were Petri nets and reliability block diagrams. Among the cited works, only Greco et al. [10] chose a layered model. Chen et al. [12] carried out a practical test. The work by Diaz et al. [21] was unique because they adopted a low-level mathematical model to evaluate its project. Our work adopted the highly representative stochastic Petri net models.

The fourth classification criterion is to verify whether the work **considered energy use** in the architecture. Our work considers the use of energy for the hospital’s internal functioning. The fifth classification criterion was the **sensitivity analysis**. Sensitivity analysis allows us to discover which components are more important to each system metric. The table shows that articles measuring availability often use this type of analysis. Availability is typically used in conjunction with sensitivity analysis as it points to the component that can be improved in the architecture. Our work uses sensitivity analysis to help with the availability and reliability metrics and to help with the component rejuvenation technique.

The sixth classification criterion was **availability**. Araujo(a) et al. [13] used the availability metric to help measure the main metric, without exposing the results of the metric itself. The seventh criterion was **reliability**. The papers that performed a reliability analysis used it as the main metric with availability. Furthermore, again, the work by Araujo(a) et al. [13] considered the reliability to measure the main metric but did not show the reliability results. The last criterion was **rejuvenation**. This work was the only one surveyed that applied rejuvenation techniques while joining the context of smart hospitals with energy and verified their impact on system availability.

## 3. Architecture Overview

**Figure 1:**
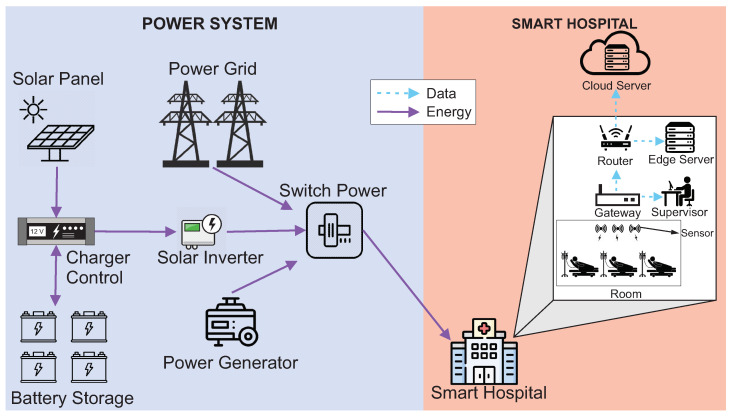
Proposed architecture of a smart hospital system.

This section presents the characteristics of the modeled architecture. Figure 1 presents the IoMT architecture adopted in this paper. The architecture has been divided into two parts to make it easier to organize and understand. The Power System represents the entire power supply for the hospital. Smart Hospital represents the hospital itself and its internal components.

The Power System has three energy sources: the Power Grid, Power Generator, and Solar Panel System. The Power Grid represents the public energy offered by public or private companies that, normally, have no connection with the hospital. The Power Generator is a power generator that normally runs on diesel and is usually used in most hospitals for eventual power outages. The photovoltaic system consists of a solar panel, charger control, battery storage, solar inverter, and switch power. The Solar Panel represents one of the photovoltaic system sources that transforms the thermal energy generated by the sun into electrical energy. Battery Storage is a battery system that stores the produced solar energy. The batteries used to store solar energy are still considered limited nowadays [25]. Thus, batteries keep the hospital supplied for a short period until the main power is restored. The Charger Control regulates the amount of energy from the solar panel to the batteries. The Solar Inverter transforms the energy generated from the direct current to the alternating current. Switch Power is an actuator that decides which energy source should be used: solar, public/private power grid, or generator. Finally, the system works, considering that energy comes primarily from solar panels. The Power Grid will be used if solar power fails. The generator will be activated when the other two resources fail.

Inside the Smart Hospital, some components will be used to monitor patients. These components distribute information across the other components. The hospital has several rooms with sensors that capture patient data from their beds. The sensors are connected to the Gateway. The Gateway collects information from all sensors and transmits it to the Router and Supervisor. The Supervisor is responsible for observing and analyzing patient data in case of an emergency. The Router will distribute the Gateway data to an edge server located in the hospital and a remote cloud server. The edge server will store the data locally at the hospital for reporting and any future queries. The cloud Server will store the data remotely to have a backup copy of the data, mainly to monitor a patient remotely. Finally, all internal components of the hospital need the energy to be available for as long as possible, and the cloud server is located at a remote place.

*Operational configurations:* To investigate the impact of energy resource redundancy and server rejuvenation on the smart hospital’s dependability, we consider three different configurations, as shown in Table 2. Configuration *A* represents a conventional smart hospital, including a power grid and a generator. Configuration *B* represents a smart hospital with a sustainable energy system. Configuration *C* reinforces configuration *B* against software aging problems running on cloud/edge servers. Configuration *C* does not involve an additional physical component; instead, a server rejuvenation mechanism at the cloud and edge, along with two logical components that control the rejuvenation process, is taken into account. The Rejuvenation Trigger is responsible for managing rejuvenation, while the Peakhour Indicator manages the rejuvenation together with the Rejuvenation Trigger if there is a peak hour policy for the hospital. Configuration *B* involves the components of the photovoltaic system, including the Solar Panel, Battery System, Charger Control, Solar Inverter, and Switch Power.

## 4. Proposed SPN Models

In this section, availability and reliability SPN models are developed following the considered operational configurations of the smart hospital’s computing infrastructure.

### 4.1. Availability Models

This subsection presents the three SPN models focused on availability calculation.

#### 4.1.1. Model A: Base Architecture with Basic Power Source

**Figure 2:**
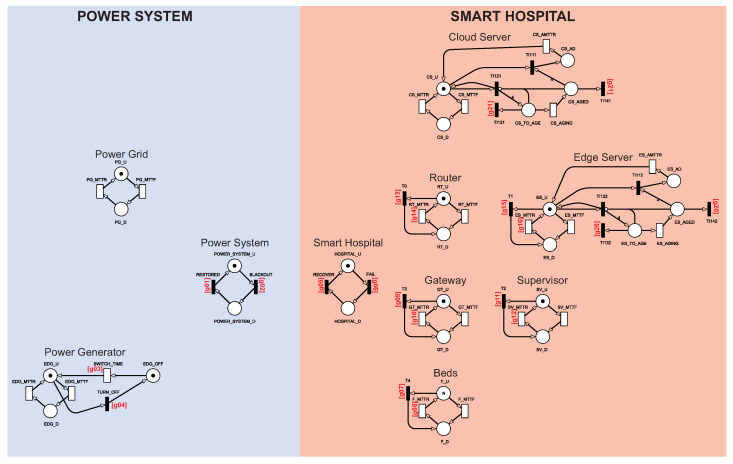
SPN model A.

Figure 2 shows model A. Model A corresponds to configuration A presented in the last section. Therefore, model A does not consider the photovoltaic system. This model represents a more common power source architecture for hospitals in general [26,27,28]. Model A will serve as a base model to compare with the following models presented in this section. The hospital is in operative mode in model A when all the internal components are active and when one of the external energy components is functioning. Two building blocks centralize the control of the two sides of the architecture using guard expressions, Power System, and Smart Hospital. Such control centralization enabled us to calculate the availability only focused on these two building blocks. All transitions with a red index mark are configured with a guard expression. Table 3 summarizes all guard expressions.

**Figure 3:**
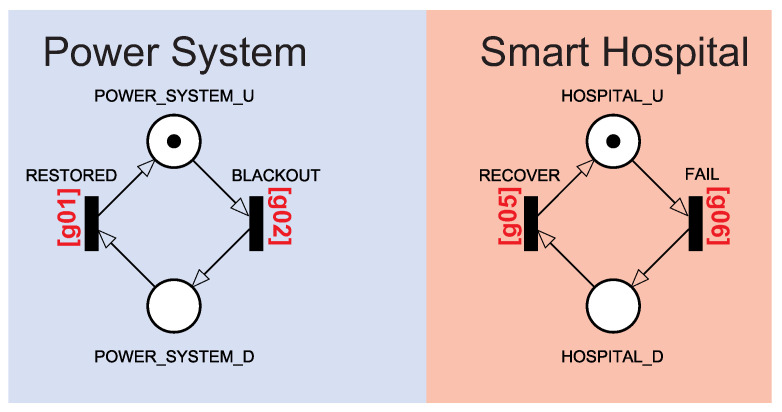
Power System and Smart Hospital building block control components.

Figure 3 shows the Power System and Smart Hospital building block components in more detail. The Power System building block component represented the system’s power status. The Power System is considered up when there is a token at POWER_SYSTEM_U. The Power System is not working when there is a token at POWER_SYSTEM_D. The change between active and inactive states is controlled by BLACKOUT and RESTORED transitions. BLACKOUT and RESTORED transitions have guard expressions. The BLACKOUT transition is activated when all energy sources are inactive, given by expression g01=(#PG_U>0)OR(#EDG_U>0). The RESTORED transition is activated when there is at least one power source active, given by expression g02=(#PG_U<1)AND(#EDG_U<1).

The Smart Hospital building block component represents the hospital system status. The hospital is active when all the Smart Hospital components are active. The hospital is deactivated when at least one of the components has tokens in the inactive state. The Smart Hospital is up when there is a token HOSPITAL_U. The Smart Hospital is not working when there is a token in HOSPITAL_D. The change between active and inactive states is caused by the transitions FAIL and RECOVER. Thus, the FAIL transition is activated when all energy sources are inactive, given by expression g05=((#CS_U>0)AND(#ES_U>0)AND(#RT_U>0)AND(GT_U>0)AND(F_D=0)AND(SV_U>0))AND(POWER_SYSTEM_U>0). The RECOVER transition is activated when there is at least one energy source active, given by expression g06=(#CS_U<1)OR(#ES_U<1)OR(#RT_D>0)OR(GT_D>0)OR(F_D>0)OR(SV_D>0).

**Figure 4:**
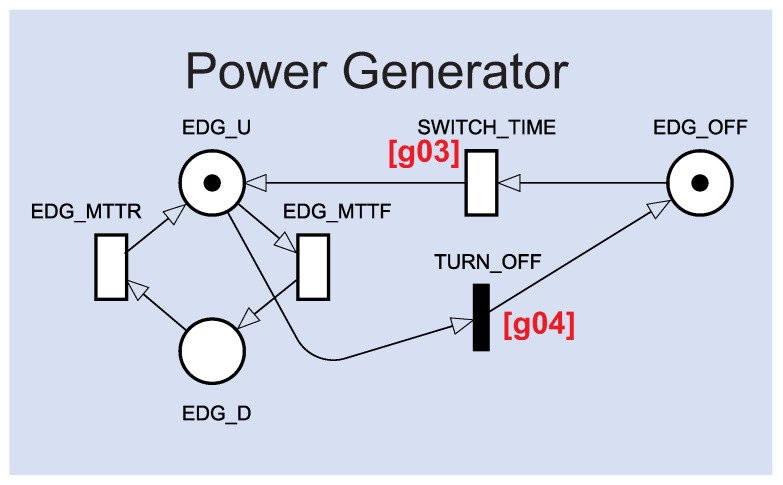
Power Generator component.

Figure 4 shows the Power Generator component in more detail. The power generator behaves differently from the other model components. The power generator has a cold standby mechanism [29]; that is, it will be only active when a certain condition is reached. The SWITCH_TIME transition will ensure the power generator is only activated when there is no other active energy source. SWITCH_TIME is supported by the guard expression: g03=((PS_U<1)OR(#IN_U<1)OR(#CC_U<1)OR((#SP_U<1)AND(#BS_U<1)))AND(#PG_U<1). The TURN_OFF transition will ensure that the power generator will be immediately deactivated when any other energy source start working again through the expression g04=((PS_U>0)AND(#IN_U>0)AND(#CC_U>0)AND((#SP_U>0)OR(#BS_U>0)))OR(#PG_U>0)

**Figure 5:**
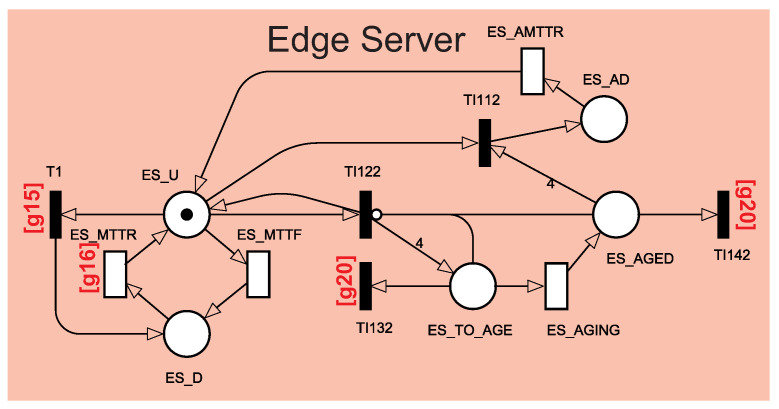
Software aging mechanism.

Figure 5 shows the aging mechanism that was used in detail. Software aging is modeled on the edge server and cloud server through a four-phase Erlang subnet [30]. The number of phases depends on the parameters obtained for each component’s aging. The use of four aging phases is based on the previous study of Melo et al. [30]. The Erlang subnet can represent software aging by increasing the [31] failure rate. When the immediate transition TI122 triggers, four tokens (four phases) move to the ES_TO_AGE place, and another token moves back to ES_U, keeping the system alive and aging on the Erlang subnet. When the timed transition ES_AGING triggers, a token moves from ES_TO_AGE to ES_AGED, representing the end of one of the aging phases. When ES_AGED has all four tokens, the immediate transition TI112 triggers, moving all tokens to ES_AD, putting the system on aging failure.

The sum of the four phases of ES_AGING represents the total time for server aging. When the timed transition ES_AMTTR triggers, the system is repaired and becomes active again. The immediate transitions TI132 and TI142 “clean up” the aging, removing the tokens from ES_TO_AGE and ES_AGED. The aging cleansing process occurs when the system fails or starts the rejuvenation process. The rejuvenation process will be presented in Section 4.1.3. The immediate transition T1 shuts down the edge server when the Power System is unavailable due to failure. When T1 fires, a token moves from ES_U to ES_D, putting the edge server in a failed state. The same behaviors happen to the cloud server.

As the metric assumes that the Power System and Smart Hospital building blocks must be in an active state for availability, different fault tolerance levels can be configured by changing their guard expressions. For example, the hospital can be considered active if at least one server or N of the M beds is active. We consider that all internal components must be active for the hospital to be available.

#### 4.1.2. Model B: Model with Photovoltaic System Inclusion

**Figure 6:**
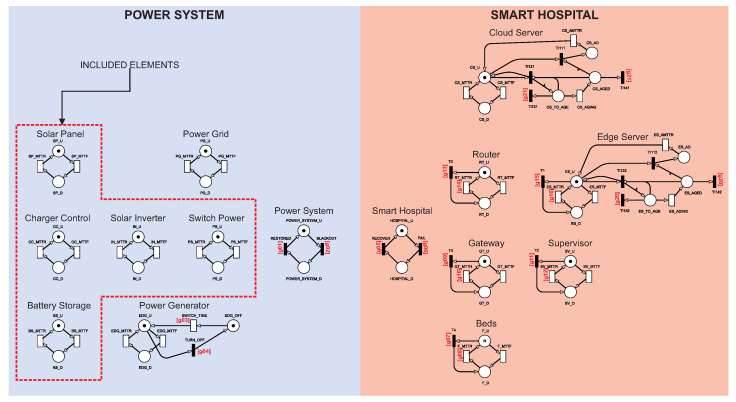
SPN model B.

Figure 6 shows model B. Model B considers the Power System and the Smart Hospital. For model B, the Power System will be improved by adding a photovoltaic system. All components are organized as they appear in the architecture for easy visualization. The components have the following characteristics: (i) Sensors are attached to patients in beds that collect a patient’s vital information and data; (ii) Patient information is distributed to the Supervisor and edge/cloud servers (for storage and monitoring); (iii) the Solar Panel will generate energy by receiving sunlight; (iv) Charger Control must adjust the level of charge that is sent to the batteries and the Solar Inverter; (v) Battery Storage must store energy to support the Solar Panel if it is not enough, and in the last case, it must sustain the hospital for a brief period that is sufficient to repair the Solar Panel; (vi) the Solar Inverter converts direct current energy (which is used by the solar panel and batteries) to alternating current, which is used by the hospital devices; (vii) Switch Power must choose which energy source to assume when another source fails; (viii) the Power Generator will only be activated if all other possible power sources fail.

For model B, the Smart Hospital part and the Smart Hospital building block component will work the same way as in model A. The entire photovoltaic system becomes part of the Power System for model B. The g01 and g02 guard expressions have been changed to update the functioning of the Power System building block component. Table 4 presents the guard expressions that have been changed for model B. The Power System building block component now considers that the internal components of the photovoltaic system need to be working. The g01 guard expression was updated to g01=((PS_U>0)AND(#IN_U>0)AND(#CC_U>0)AND((#SP_U>0)OR(#BS_U>0)))OR(#PG_U>0)OR(#EDG_U>0). The g02 guard expression was also updated to g02=((PS_U<1)OR(#IN_U<1)OR(#CC_U<1)OR((#SP_U<1)AND(#BS_U<1)))AND(#PG_U<1)AND(#EDG_U<1) to cover the new behaviors of the photovoltaic system.

Finally, as already mentioned, the Power System and Smart Hospital building block components serve to centralize the status of each part of the architecture and simplify the metric equations (availability and downtime). P represents the probability, and # represents the number of tokens in a given place. The downtime (D) can be obtained by Equation (1), where A represents the system availability and 8760 the number of hours in the year. The equation that calculates availability for all models is given by Equation (2), that is, the probability that the Power System and Smart Hospital are both working at the same time. It is noteworthy that the user can change the guard expression of the central building blocks for other configurations.

(1) D=(1−A)×8760

(2) A=P{(#HOSPITAL_U>0)AND(#POWER_SYSTEM_U>0)}

#### 4.1.3. Model C: Model B with Rejuvenation Technique Inclusion

**Figure 7:**
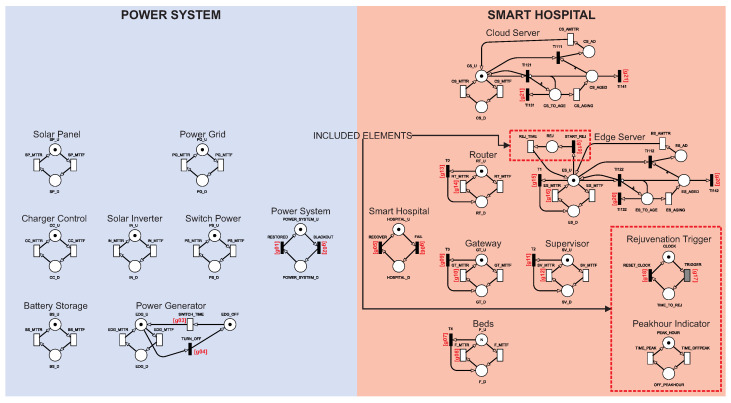
SPN model C for availability.

Software rejuvenation is a technique that usually improves system availability [32]. Rejuvenation has the function of preventing crashes and reducing performance. The technique involves occasionally stopping the software from running to clear its state and restarting it [33]. Figure 7 shows model C, which extends model B. The extension is based on the rejuvenation process under the cloud and edge servers. A Rejuvenation Trigger component represents peak times. The other energy and hospital components work the same as in model B. Rejuvenation introduces planned interruptions. It can be advantageous to differentiate the peak and off-peak periods to plan the [33] rejuvenation strategy. We consider peak hours as day periods and off-peak hours as night periods (12 h each). The peak component has two locations called PEAK_HOUR and OFFPEAK_HOUR PEAK_HOUR and OFFPEAK_HOUR represent peak hours and off-peak hours, respectively. The peak component also has two timed transitions. When TIME_PEAK is triggered, it puts the system in peak hours. When TIME_OFFPEAK is triggered, the system goes to off-peak hours.

The sensitivity analysis presented later pointed out the edge server as the most sensitive component. Thus, rejuvenation was applied to the edge server since many studies prove that servers suffer from software aging, which impacts system availability [32,34,35]. The rejuvenation model was based on the model proposed by Garg et al. [36]. ES_U represents that the edge server is active. ES_D indicates that the edge server has failed and is disabled. When the ES_MTTF transition is triggered, a token moves from ES_U to ES_D, putting the edge server in a failed state. When the ES_MTTR transition is triggered, the edge server is repaired, putting it back into the active state. The remaining component was explained in Section 4.1.1.

Another component presented in model C is the Rejuvenation Trigger, which is when the edge server will rejuvenate. The aging and rejuvenation process was based on the model proposed by [37]. The Clock place is used to store the edge server rejuvenation token. When the TRIGGER transition is triggered, a token moves from CLOCK to TIME_TO_REJ, indicating that the system is ready to begin rejuvenation. The TRIGGER represents the time-until-rejuvenation, i.e., the time interval until the rejuvenation process occurs again. When TIME_TO_REJ has a token, the immediate transition START_REJ triggers, moving a token from ES_U to REJ, putting the system in a rejuvenation state. When REJ has a token, the immediate T0 transition triggers, moving the token from TIME_TO_REJ to CLOCK, starting the countdown for the next rejuvenation. When REJ has a token, the immediate transitions TI132 and TI142 clear the aging phase progress. When the timed transition REJ_TIME triggers, a token moves from REJ to ES_U, rejuvenating the system. The REJ_TIME transition represents the time to rejuvenate the edge server. During the rejuvenation process, the system is unavailable. Table 5 shows some guard transitions added and modified to grant the new behaviors of the rejuvenation mechanism.

The model uses two rejuvenation policies with peak times (no-peak policy and peak policy). The no-peak policy ignores peak times. The TRIGGER transition indicates the time for the rejuvenation to occur regardless of being in a peak period or not. The peak policy prevents the system from starting rejuvenation during peak hours. When the system is off-peak (has a token in OFFPEAK_HOUR), the rejuvenation behaves the same as the no-peak policy. However, when the system is in peak hours (it has a token in PEAK_HOUR), the TRIGGER transition is not triggered even after the associated time unit has elapsed. Considering model C, the system is available when the edge server token is active (a token in ES_U. Finally, Equation (2) defines the availability of model C.

### 4.2. Reliability Model

**Figure 8:**
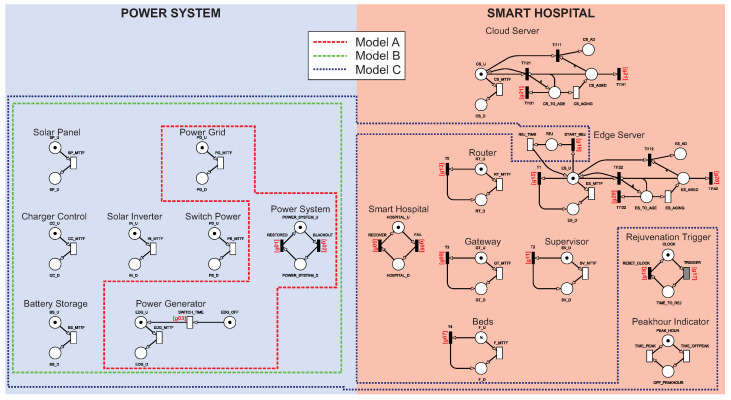
Reliability models created from availability models.

Reliability is the conditional probability of a system remaining operational over a time interval [0, t], considering that it was operational at t = 0. Figure 8 presents the reliability models of the proposed system. The three possible configurations were organized into a single figure. In principle, the model works the same as the availability SPN models. In this model, the components do not have the transitions that allow their recovery. The reliability of the presented models can be calculated using Equation (3). Finally, p calculates the probability that the system is unavailable. The equation can generate a curve that shows how reliability declines over time.

(3) R=1−(P{((#HOSPITAL_D>0)OR(#POWER_SYSTEM_D>0))})

## 5. Sensitivity Analysis

This section presents the sensitivity analysis. Table 6 shows the results for the sensitivity of the availability models’ components. The sensitivity experiment was obtained through stationary simulation. This decision was determined by the system’s significant number of possible states. Because of the numerous components, only the first ten results were selected for each model.

Model A uses the Power Grid and a diesel generator to keep the hospital active. Thus, it can be seen that the Power Grid was the most critical component of the architecture; that is, its sensitivity index causes a significant impact on system availability. Power issues impact the final availability of the system, and the Power Grid has a slightly more critical sensitivity index than the second place, which is the edge’s aging time. From the second position onward (model A), all components are internal to the hospital, as the generator is only occasionally turned on. The other two components that proved to be more critical are the edge and cloud servers due to their low failure and recovery times and their respective aging. The edge server and cloud server can be considered critical because they presented a higher sensitivity index than the other components.

Model B brings redundancy when adding the photovoltaic system, an unusual configuration in hospitals today. The Power Grid index is no longer among the ten most critical. The edge aging (ES_AGING) sensitivity index becomes more critical and takes the first position, followed by cloud aging (CS_AGING). The photovoltaic system increases system availability. However, aging is still a critical factor for availability. The cloud and edge-related components were the most critical for model B, they were also more critical than most components in model A, but their values grew from $10^{-4}$ to $10^{-3}$. The number of sensors represents a very small impact on the system, even though it appears among the ten most critical. The variation in the number of sensors did not significantly impact availability.

In addition to proposing using the photovoltaic system, model C also proposes rejuvenating the edge component. The edge server became more critical in model B, so rejuvenation was applied. The most relevant indices for this model are still around $10^{-3}$ and decrease to values in $10^{-5}$. The most critical index for this scenario is the cloud’s aging time. The edge’s aging index decreases due to the rejuvenation applied to the component. As the rejuvenation was not applied in the cloud, it remained with the same index. Edge and cloud failure and recovery components continue to occupy the top positions of the sensitivity table along with aging times. The criticality reduction in cloud and edge recovery and failure indices could be solved with some form of redundancy. However, the objective of this work was to check rejuvenation issues to make the process cheaper.

**Figure 9:**
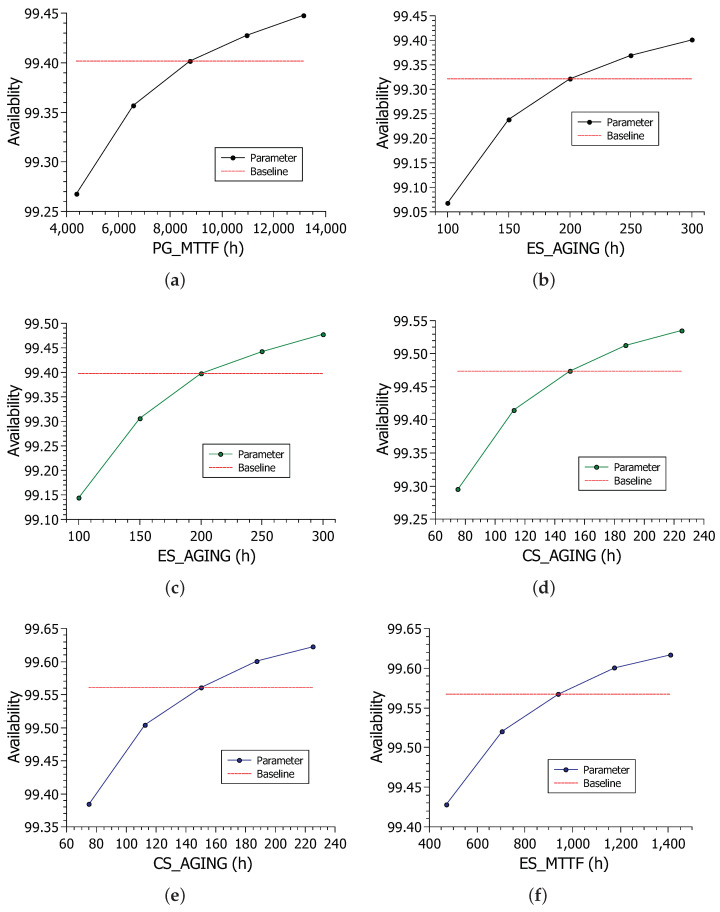
Results for the sensitivity of availability models: (**a**) PG_MTTF × model A; (**b**) ES_AGING × model A; (**c**) ES_AGING × model B; (**d**) CS_AGING × model B; (**e**) CS_AGING × model C; (**f**) ES_MTTF × model C.

Figure 6 shows the variation of the two most critical indices for each model. Figure 9a shows the impact of PG_MTTF parameter variation on system availability. Availability ranged from about 99.25% to 99.45%. As the curve presents an exponential behavior, it can be deduced that the less reliable the energy, the more abruptly the value tends to fall. Figure 9b shows the impact of edge aging in model A. The variation in the edge aging time caused the availability to vary greatly, but the maximum and average values were lower than those seen in the PG_MTTF variation. Figure 9c shows the impact of edge aging on model B availability.

Figure 9d shows the impact of cloud aging on model B. The cloud aging time variation resulted in slightly higher availability, but the edge aging had a large enough variation to surpass the cloud aging time as the most critical component. Figure 9e shows the impact of the CS_AGING parameter on the availability of model C. Values range from about 99.38% to about 99.62%. The variation is still large, given the parameter’s sensitivity index value, which is expected. However, the addition of edge rejuvenation caused the beginning and end of the sensitivity result interval to increase. The range in the previous scenarios started just below 99.30%, whereas in this scenario, it starts above 99.30%. The same behavior is repeated for the upper range, increasing more than the model B sensitivity result. Finally, Figure 9f shows the impact of the edge failure time on model C’s availability. Cloud aging had a noticeably longer range between values, so the edge recovery time was the second most important.

## 6. Case Studies

The results of different experiments with the models proposed in the previous section are presented. Table 7 presents the parameters used to feed the proposed models. The values used were taken from other validated studies. The values were taken from four papers [11,18,38,39]. The TIME_PEAK and TIME_OFFPEAK values represent daytime and nighttime, respectively. The SWITCH_TIME has been set to 0.0833333 h. Edge server and cloud server aging times were based on their MTTFs, as per the work of [37]. The experiments were performed using the Mercury modeling tool [40]. All model transitions work in single server mode, except Beds component transitions. The Beds component works in Infinite Server mode. Beds work in infinite server mode due to their nature, as the sensors work and are repaired in parallel. The availability experiment was obtained through a stationary simulation due to the model size and rejuvenation particularities. The reliability experiment was carried out through a transient simulation for the above-mentioned reasons.

### 6.1. Case Study 1—Availability and Downtime Analysis of the Three Models

**Figure 10:**
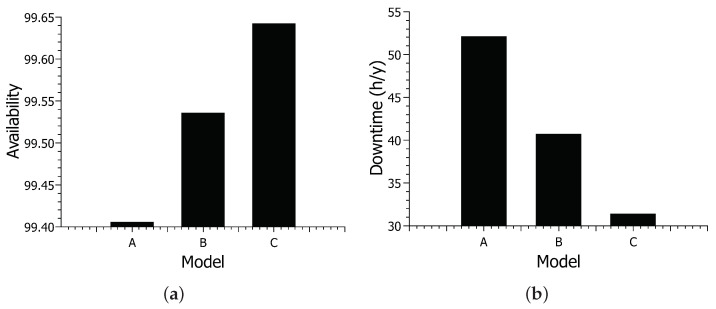
Results for availability models: (**a**) availability; (**b**) downtime.

In this section, the results of the availability model will be shown. Figure 10 shows the results for availability and downtime for different models. Figure 10a shows the results for availability. Model A had the lowest availability, with approximately 99.40% availability. With approximately 99.53% availability, model B increased about 0.13% compared to model A. Model C achieved the highest available value, with approximately 99.64% availability. Overall, there was an increase of about 0.24% between models A and C. Model C showed an increase of 0.11% compared to model B proposed in this work.

Figure 10b shows system downtime based on different Configurations. Model A showed the longest downtime, with approximately 52 h of downtime. With approximately 40 h of downtime, model B showed a reduction of about 12 h compared to model A. Model C had the lowest downtime, with around 31 h of downtime. Model C shows a difference of about 21 h concerning model A—almost a full day. The reduction in model C considering model B was about 9 h.

The results for availability vary considerably little, but looking at the downtime results, we can see that it is an obvious difference. Availability has slightly low variance, as power itself is a component that must be reliable; thus, it rarely fails. However, the inclusion of the solar energy system further increases the availability as it is a more reliable source of energy than a public one.The addition of the photovoltaic system showed the greatest increase in availability among the scenarios. Energy is indeed a component to be considered in modeling this type of system. Model C did not show an increase as big as model B, as it only adds a rejuvenating mechanism, but it is still a visible increase. Furthermore, a rejuvenation case in the cloud could increase the result of the last scenario. However, this paper has focused more on internal components in the hospital’s control.

### 6.2. Case Study 2—Analysis of Availability and Downtime of Rejuvenation Policies in Model C

**Figure 11:**
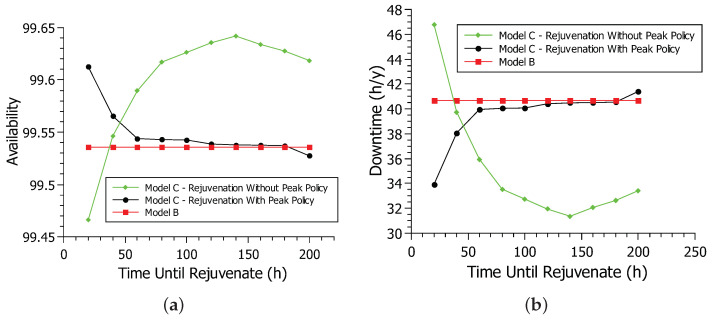
Comparison of levels of availability and downtime in relation to the rejuvenation model: (**a**) availability; (**b**) downtime.

Figure 11 shows the availability and downtime of the rejuvenation model. Again, the simulation method was used with the Mercury tool [40] considering the availability model B and the two rejuvenation policies in model C. Figure 11a shows the availability of the system for model B and model C of rejuvenation in non-peak and peak policies. The deterministic transition time TRIGGER is varied in the simulations from 20 to 200, with intervals of 20 h, based on the model proposed in [33]. When time-until-rejuvenation (TRIGGER) is at 20 h, the no-peak policy has lower availability than the extended model, at about 99.46% availability. After the time-until-rejuvenation reaches 40 h, the no-peak policy availability improves significantly, outpacing the extended model availability.

The availability of the no-peak policy continues to increase as the time-until-rejuvenation increases. When the time-until-rejuvenation was at 20 h, the rejuvenation happened so often that it hindered system availability. Too frequent rejuvenation impairs availability as the system goes down for a short period during rejuvenation. After the time-until-rejuvenation reaches 40 h or more, rejuvenation occurs less frequently. Rejuvenation prevents the system from having more serious failures and improving availability. As time-until-rejuvenation increases, availability improves, as rejuvenation occurs at more strategic times until it peaks at 140 h with an availability of 99.64%. After the time-until-rejuvenation exceeds 140 h, rejuvenation begins to take too long to occur, making the system prone to more serious failures and decreasing system availability.

The peak policy behaves differently from the no-peak policy. When the time-until-rejuvenation is at 20 h, the peak policy hits its highest availability peak at 99.61%. As time-until-rejuvenation increases, the availability of the no-peak policy decreases. The peak policy only rejuvenates if the system is off-peak, conducting far fewer rejuvenation processes than the no-peak policy. The time-until-rejuvenation in 20 h is already the ideal time for rejuvenating the peak policy. As the time-until-rejuvenation increases, rejuvenation takes a long time, impairing system availability. After exceeding 160 h until rejuvenation, the peak policy availability is lower than that of the extended model. In general, rejuvenation was able to increase system availability greatly. All non-peak and peak policy values were better than model B values, especially shorter until rejuvenating. While the no-peak policy has far better availability than the peak policy, rejuvenating during peak hours may not be the best choice. The peak policy is also a smart choice in a scenario where this cannot happen, with better results than model B, especially in 20 h until rejuvenation.

Figure 11b shows the system downtime for model B and the rejuvenation model in both peak and non-peak hour policies. This graph reflects the availability graph, showing downtime over a year. When the time-until-rejuvenation is at 20 h, the no-peak policy is at its best downtime of 33.92 h. As time-until-rejuvenation increases, downtime also increases. The lower the availability, the greater the downtime. After the time-until-rejuvenation passes 180 h, the peak policy’s downtime reaches 41.30 h, surpassing the 40.68 h of model B. The no-peak policy has the longest downtime at 46.78 h when the time until rejuvenation is 20 h. However, downtime decreases as the time-until-rejuvenation increases until 31.36 h per year when the time-until-rejuvenation is 140 h. After 140 h until rejuvenation, the downtime starts to increase, reaching 33.42 h when the time-until-rejuvenation is 200 h. Therefore, the two rejuvenation policies had a lower downtime than model B, especially when each policy’s time-until-rejuvenation is at its peak.

### 6.3. Case Study 3—Analysis of the Impact of Time-Until-Rejuvenation and Edge MTTF on Availability

**Figure 12:**
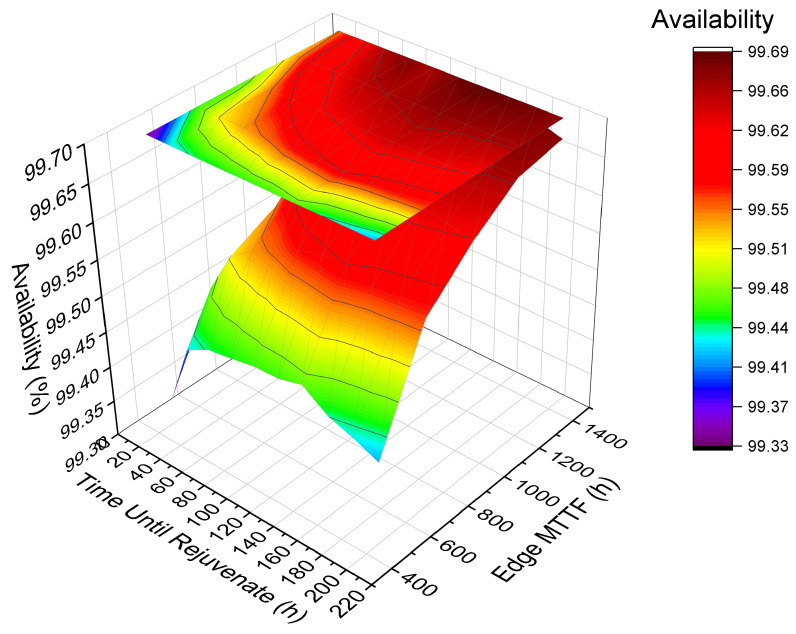
Availability analysis by varying concomitant factors.

Figure 12 presents a 3D surface graph to show the system’s behavior considering availability by varying two factors with a high impact on availability. Edge MTTF and time-until-rejuvenation factors were varied in five ranges: base value, minus 25%, minus 50%, plus 25%, and 50%. Colors are related to the result of availability. The bar on the right indicates the magnitude of the results. The upper part of the bar indicates the highest availabilities, while the lower part indicates the lowest availabilities achieved. Thus, the red color means the highest availability, and the purple color means the lowest availability. Changing the MTTF is more impactful than changing the time-until-rejuvenation. The color red is present in most of the Edge MTTF projections. If the MTTF value is low, the availability always tends to be low, even changing the time-until-rejuvenation. Therefore, the result indicates that increasing the MTTF improves system availability. However, finding an ideal value for time-until-rejuvenation will also benefit the system’s availability.

### 6.4. Case Study 4—Reliability Analysis

**Figure 13:**
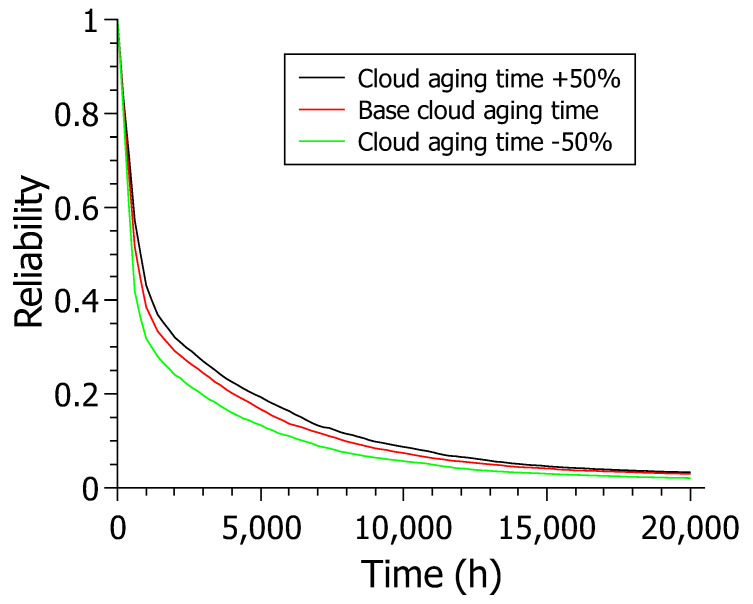
Reliability results varying the CS_AGING parameter in model C.

This section displays the results for the model’s reliability metric. Figure 13 shows the results for reliability over 20,000 h varying the cloud aging time (CS_AGING) for model C. The cloud aging time was chosen due to its high sensitivity index. There are three reliability models. However, varying model C proved more relevant when analyzing the sensitivity analysis results. The cloud does not have a rejuvenation mechanism. Thus, the cloud became the most critical component system. The experiment simulates a reliability analysis in scenarios where the cloud cannot be rejuvenated, and a more or less reliable cloud must be chosen. All results decrease over time, as this is the nature of this experiment. In other words, the reliability of a system always tends to decrease over time.

The scenario where the cloud’s aging time is reduced by 50% presented values that remain below the values presented in the other two scenarios.The base scenario also touches the X-axis at values above 20,000 h. The scenario where the cloud has its aging time increased by 50% shows better results than all other scenarios, staying above the others from the start and following through for practically the entire time interval.

The +50% scenario also touches the X-axis at values greater than the 20,000 h of the experiment as in the other scenarios. The +50% scenario remains above the other scenarios at the 20,000 h endpoint on the chart, with around 3% of confidence versus around 2% for the other two scenarios. The results show a trend towards increased reliability in obtaining a slower aging cloud, which is expected. The main point of this case study is that, given a budget, it is feasible to select the most advantageous cloud for the company as long as it meets the established dependability criteria. For example, if there is a need for a cloud that makes the system at least 20% reliable after 3000 h, the base cloud would be enough to meet the demand without investing more in a more expensive cloud.

## 7. Conclusions and Future Works

This paper proposed stochastic Petri net models for an architecture of intelligent hospitals to help system administrators plan computing architectures. The models consider several factors that influence the total availability of the system. Energy is one of the major factors considered, and the use of solar energy, in addition to making the hospital more sustainable, showed a considerable increase in availability. The aging of more complex components, such as servers, has also had a major impact on system availability. The use of rejuvenation to treat aging was considered in the model, and the analysis shows that the model can estimate the ideal time between rejuvenation for a given model. The availability model can be configured using about 20 different parameters, while the reliability model has about half that amount.

Models provide accurate estimates of availability, downtime, and reliability metrics. The models were demonstrated by carrying out four case studies, one of them for the reliability analysis and the other three for further availability analyses. The results show how each model behaves with varyied parameters by sensitivity analyses. The case studies provide a practical guide that shows how a system administrator can apply the model to perform assessments of various configurations for a sustainable smart hospital architecture.

This work presents some limitations that are important to highlight: (i) Aiming to avoid the “state-space explosion” problem, a couple of simplifications were necessary for the models. There are complex components such as the power grid and cloud server, for example, that are treated as encapsulated components with single corresponding MTTF/MTTR parameters. (ii) The availability is sometimes influenced by user interaction, security risks, and environmental issues. None of these aspects were investigated, only the aging processes. (iii) The rejuvenation strategy was applied only at the cloud/edge servers. However, the other hardware equipment (router, supervisor, and gateway) may also suffer from aging. We did not consider that. (iv) We have used only heterogeneous redundancy (distinct power supply sources) at the power system side. The hospital can have, for example, more than one power diesel generator, but we did not consider such a possibility. Future work intends to carry out a performance analysis to verify the impact that the availability of components can have on the response time and throughput of the system. More external factors can also be considered, such as the functioning of the photovoltaic system on cloudy days.

## Figures and Tables

**Table 1:** Related Works.

Work	Context	Metrics	Evaluation Method	Consider the Use of Energy	Sensitivity Analysis	Availability	Reliability	Rejuvenation
[9]	Smart Healthcare and IoT	Patient Length of Stay (LoS), Resource Utilization, and Average Patient Waiting Time	Petri Net	No	No	No	No	No
[17]	Smart Healthcare and IoT	Availability	Stochastic Petri Net	No	No	Yes	No	No
[10]	IoT	Resource Utilization	Layered Model	No	No	No	No	No
[12]	Smart Healthcare	QoS	Practical Test	No	No	No	No	No
[13]	Smart Healthcare	Message Delivery Probability	Stochastic Petri Net	No	No	Yes	No	No
[19]	Smart Healthcare	Availability and Downtime	Stochastic Petri Net	No	Yes	Yes	No	No
[20]	Smart Healthcare	Availability, Throughput, and Service Time	Stochastic Petri Net	No	Yes	Yes	No	No
[18]	Smart Healthcare	Mean Response Time, Resource Utilization, Discard, Availability, and Downtime	Stochastic Petri Net	No	Yes	Yes	No	No
[11]	Smart Building and Energy	Availability and Energy Cost	Stochastic Petri Net	Yes	No	Yes	No	No
[21]	Energy	Reliability	Mathematical Model	Yes	No	No	Yes	No
[22]	Energy	Availability and Reliability	Reliability Block Diagram	Yes	No	Yes	Yes	No
[23]	Energy	Availability and Reliability	Reliability Block Diagram	Yes	No	Yes	Yes	No
[24]	Energy	Availability and Reliability	Framework	Yes	No	Yes	Yes	No
This Work	Energy and Smart Healthcare	Availability and Reliability	Stochastic Petri Net	Yes	Yes	Yes	Yes	Yes

**Table 2:** Operational configurations.

Config.	Smart Hospital	Power Grid	Generator	Photovoltaic System	Rejuvenation Trigger	Peakhour Indicator
A	X	X	X			
B	X	X	X	X		
C	X	X	X	X	X	X

**Table 3:** Guard expressions for model A transitions.

Guard Index	Guard Expression
g01	(#PG_U>0) OR (#EDG_U>0)
g02	(#PG_U<1) AND (#EDG_U<1)
g03	((PS_U<1) OR (#IN_U<1) OR (#CC_U<1) OR ((#SP_U<1) AND (#BS_U<1))) AND (#PG_U<1)
g04	((PS_U>0) AND (#IN_U>0) AND (#CC_U>0) AND ((#SP_U>0) OR (#BS_U>0))) OR (#PG_U>0)
g05	((#CS_U>0) AND (#ES_U>0) AND (#RT_U>0) AND (GT_U>0) AND (F_D=0) AND (SV_U>0)) AND (POWER_SYSTEM_U>0)
g06	(#CS_U<1) OR (#ES_U<1) OR (#RT_D>0) OR (GT_D>0) OR (F_D>0) OR (SV_D>0)
g07	(#POWER_SYSTEM_D>0)
g08	(#POWER_SYSTEM_U>0)
g09	(#POWER_SYSTEM_D>0)
g10	(#POWER_SYSTEM_U>0)
g11	(#POWER_SYSTEM_D>0)
g12	(#POWER_SYSTEM_U>0)
g13	(#POWER_SYSTEM_D>0)
g14	(#POWER_SYSTEM_U>0)
g15	(#POWER_SYSTEM_D>0)
g16	(#POWER_SYSTEM_U>0)
g20	(#ES_D>0)
g21	(#CS_D>0)

**Table 4:** New guard expressions for the model B transitions.

Guard Index	Guard Expression
g01	((PS_U>0) AND (#IN_U>0) AND (#CC_U>0) AND ((#SP_U>0) OR (#BS_U>0))) OR (#PG_U>0) OR (#EDG_U>0)
g02	((PS_U<1) OR (#IN_U<1) OR (#CC_U<1) OR ((#SP_U<1) AND (#BS_U<1))) AND (#PG_U<1) AND (#EDG_U<1)

**Table 5:** New guard expressions for the model C transitions.

Guard Index	Guard Expression
g17 *	(OFFPEAK_HOUR>0)
g18	(#REJ>0)
g19	(#TIME_TO_REJ>0)
g20	(#REJ>0) OR (#ES_D>0)

* Only used if there is a peak hour policy.

**Table 6:** Sensitivity analysis results for the 3 availability models.

Model A		Model B		Model C	
**Component**	**Index**	**Component**	**Index**	**Component**	**Index**
PG_MTTF	8.80 × 10${}^{-4}$	ES_AGING	3.36 × 10${}^{-3}$	CS_AGING	2.39 × 10${}^{-3}$
ES_AGING	8.18 × 10${}^{-4}$	CS_AGING	2.42 × 10${}^{-3}$	ES_MTTF	1.90 × 10${}^{-3}$
ES_MTTF	7.64 × 10${}^{-4}$	ES_MTTR	2.37 × 10${}^{-3}$	CS_MTTR	1.87 × 10${}^{-3}$
ES_MTTR	7.53 × 10${}^{-4}$	CS_MTTR	1.88 × 10${}^{-3}$	CS_MTTF	1.51 × 10${}^{-3}$
CS_AGING	6.06 × 10${}^{-4}$	ES_MTTF	1.48 × 10${}^{-3}$	ES_AGING	1.39 × 10${}^{-3}$
CS_MTTR	4.38 × 10${}^{-4}$	CS_MTTF	1.45 × 10${}^{-3}$	ES_MTTR	1.00 × 10${}^{-3}$
CS_MTTF	4.19 × 10${}^{-4}$	GT_MTTF	6.31 × 10${}^{-5}$	TIMECLOCK	1.05 × 10${}^{-4}$
SW_MTTR	1.93 × 10${}^{-4}$	SW_MTTF	5.73 × 10${}^{-5}$	SW_MTTR	6.53 × 10${}^{-5}$
GT_MTTR	1.59 × 10${}^{-4}$	N	2.79 × 10${}^{-5}$	SU_MTTF	6.24 × 10${}^{-5}$
SE_MTTR	3.48 × 10${}^{-5}$	CC_MTTF	2.20 × 10${}^{-5}$	PG_MTTF	6.10 × 10${}^{-5}$

**Table 7:** Input parameters for proposed models.

Availability Parameters		
Component	MTTF (Hours)	MTTR (Hours)
Sensors/Actuators	300,000	1
Gateway	480,770	8
Supervisor	44,957	1
Router	698,220	8
Cloud Server	760	0.74
Edge Server	940	1.37
Solar Panel	219,000	8
Battery System	47,829	8
Charge Control	70,080	8
Solar Inverter	24,820	8
Emergency Diesel Generator	636	37
Power Grid	8757	4.807
**Rejuvenation Parameters**		
Transition	Time (Hours)	
TRIGGER	20–200 (steps od 20)	
REJ_TIME	0.0333333	
ES_AGING	200	
CS_AGING	150	
TIME_OFFPEAK	12	
TIME_PEAK	12	

## Data Availability

Not applicable.

## References

[1] Nguyen T.A., Fe I., Brito C., Kaliappan V.K., Choi E., Min D., Lee J.W., Silva F.A. (2021). Performability Evaluation of Load Balancing and Fail-over Strategies for Medical Information Systems with Edge/Fog Computing Using Stochastic Reward Nets. Sensors.

[2] Santos L., Cunha B., Fé I., Vieira M., Silva F.A. (2021). Data Processing on Edge and Cloud: A Performability Evaluation and Sensitivity Analysis. J. Netw. Syst. Manag..

[3] Said A.M., Yahyaoui A., Abdellatif T. (2021). Efficient Anomaly Detection for Smart Hospital IoT Systems. Sensors.

[4] Zou X., Dai Y.S., Doebbeling B., Qi M. (2007). Dependability and security in medical information system. Lecture Notes in Computer Science (Including Subseries Lecture Notes in Artificial Intelligence and Lecture Notes in Bioinformatics).

[5] Gardner R.M., Allan Pryor T., Warner H.R. (1999). The HELP hospital information system: Update 1998. Int. J. Med. Inform..

[6] Nelson N.C. (2007). Downtime procedures for a clinical information system: A critical issue. J. Crit. Care.

[7] Jenkins D., Qureshi R.S., Moinudheen J., Pathan S.A., Thomas S.H. (2020). Evaluation of electronic medical record downtime in a busy emergency department. Qatar Med. J..

[8] Coffey P.S., Postal S., Houston S.M., McKeeby J.W. (2016). Lessons Learned from an Electronic Health Record Downtime. Perspect. Health Inf. Manag..

[9] Oueida S., Kotb Y., Aloqaily M., Jararweh Y., Baker T. (2018). An edge computing based smart healthcare framework for resource management. Sensors.

[10] Greco L., Ritrovato P., Xhafa F. (2019). An edge-stream computing infrastructure for real-time analysis of wearable sensors data. Future Gener. Comput. Syst..

[11] Araujo E., Pereira P., Dantas J., Maciel P. (2021). Dependability Impact in the Smart Solar Power Systems: An Analysis of Smart Buildings. Energies.

[12] Chen M., Li W., Hao Y., Qian Y., Humar I. (2018). Edge cognitive computing based smart healthcare system. Future Gener. Comput. Syst..

[13] Araujo J., Silva B., Oliveira D., Maciel P. Dependability evaluation of a mhealth system using a mobile cloud infrastructure. Proceedings of the 2014 IEEE International Conference on Systems, Man, and Cybernetics (SMC).

[14] Carvalho D., Rodrigues L., Endo P.T., Kosta S., Silva F.A. Mobile Edge Computing Performance Evaluation using Stochastic Petri Nets. Proceedings of the 2020 IEEE Symposium on Computers and Communications (ISCC).

[15] Carvalho D., Rodrigues L., Endo P.T., Kosta S., Silva F.A. (2020). Edge servers placement in mobile edge computing using stochastic Petri nets. Int. J. Comput. Sci. Eng..

[16] Silva F.A., Rodrigues M., Maciel P., Kosta S., Mei A. Planning mobile cloud infrastructures using stochastic petri nets and graphic processing units. Proceedings of the 2015 IEEE 7th International Conference on Cloud Computing Technology and Science (CloudCom).

[17] Santos G.L., Gomes D., Kelner J., Sadok D., Silva F.A., Endo P.T., Lynn T. (2020). The internet of things for healthcare: Optimising e-health system availability in the fog and cloud. Int. J. Comput. Sci. Eng..

[18] Rodrigues L., Gonçalves I., Fé I., Endo P.T., Silva F.A. (2021). Performance and availability evaluation of an smart hospital architecture. Computing.

[19] Da Silva Lisboa M.F.F., Santos G.L., Lynn T., Sadok D., Kelner J., Endo P.T. Modeling the availability of an e-health system integrated with edge, fog and cloud infrastructures. Proceedings of the 2018 IEEE Symposium on Computers and Communications (ISCC).

[20] Santos G.L., Endo P.T., da Silva Lisboa M.F.F., da Silva L.G.F., Sadok D., Kelner J., Lynn T. (2018). Analyzing the availability and performance of an e-health system integrated with edge, fog and cloud infrastructures. J. Cloud Comput..

[21] Diaz P., Egido M.A., Nieuwenhout F. (2007). Dependability analysis of stand-alone photovoltaic systems. Prog. Photovolt. Res. Appl..

[22] Collins E., Dvorack M., Mahn J., Mundt M., Quintana M. Reliability and availability analysis of a fielded photovoltaic system. Proceedings of the 2009 34th IEEE Photovoltaic Specialists Conference (PVSC).

[23] Sayed A., El-Shimy M., El-Metwally M., Elshahed M. (2019). Reliability, availability and maintainability analysis for grid-connected solar photovoltaic systems. Energies.

[24] Cai B., Liu Y., Ma Y., Huang L., Liu Z. (2015). A framework for the reliability evaluation of grid-connected photovoltaic systems in the presence of intermittent faults. Energy.

[25] Hassan M., Saha S., Haque M.E. (2021). A framework for the performance evaluation of household rooftop solar battery systems. Int. J. Electr. Power Energy Syst..

[26] Kolokotsa D., Tsoutsos T., Papantoniou S. (2012). Energy conservation techniques for hospital buildings. Adv. Build. Energy Res..

[27] Yoshida S., Ito K., Yokoyama R. (2007). Sensitivity analysis in structure optimization of energy supply systems for a hospital. Energy Convers. Manag..

[28] Ji R., Qu S. (2019). Investigation and Evaluation of Energy Consumption Performance for Hospital Buildings in China. Sustainability.

[29] Araújo G., Rodrigues L., Oliveira K., Fé I., Khan R., Silva F.A. (2021). Vehicular cloud computing networks: Availability modelling and sensitivity analysis. Int. J. Sens. Netw..

[30] Melo M., Maciel P., Araujo J., Matos R., Araujo C. Availability study on cloud computing environments: Live migration as a rejuvenation mechanism. Proceedings of the 2013 43rd Annual IEEE/IFIP International Conference on Dependable Systems and Networks (DSN).

[31] Machida F., Kim D.S., Trivedi K.S. (2013). Modeling and analysis of software rejuvenation in a server virtualized system with live VM migration. Perform. Eval..

[32] Matos R., Araujo J., Alves V., Maciel P. Experimental evaluation of software aging effects in the eucalyptus elastic block storage. Proceedings of the 2012 IEEE International Conference on Systems, Man, and Cybernetics (SMC).

[33] Wang D., Xie W., Trivedi K.S. (2007). Performability analysis of clustered systems with rejuvenation under varying workload. Perform. Eval..

[34] Huang Y., Kintala C., Kolettis N., Fulton N.D. Software rejuvenation: Analysis, module and applications. Proceedings of the Twenty-Fifth International Symposium on Fault-Tolerant Computing, Digest of Papers.

[35] Torquato M., Araujo J., Umesh I., Maciel P. (2018). Sware: A methodology for software aging and rejuvenation experiments. J. Inf. Syst. Eng. Manag..

[36] Garg S., Puliafito A., Telek M., Trivedi K.S. Analysis of software rejuvenation using Markov regenerative stochastic Petri net. Proceedings of the Sixth International Symposium on Software Reliability Engineering, ISSRE’95.

[37] Torquato M., Umesh I., Maciel P. (2018). Models for availability and power consumption evaluation of a private cloud with VMM rejuvenation enabled by VM Live Migration. J. Supercomput..

[38] Marqusee J., Ericson S., Jenket D. (2020). Emergency Diesel Generator Reliability and Installation Energy Security.

[39] Perdue M., Gottschalg R. (2015). Energy yields of small grid connected photovoltaic system: Effects of component reliability and maintenance. IET Renew. Power Gener..

[40] Silva B., Matos R., Callou G., Figueiredo J., Oliveira D., Ferreira J., Dantas J., Lobo A., Alves V., Maciel P. Mercury: An integrated environment for performance and dependability evaluation of general systems. Proceedings of the Industrial Track at 45th Dependable Systems and Networks Conference, DSN.

